# Recurrent Mutations of the Active Adenylation Domain of UBA1 in Atypical Form of VEXAS Syndrome

**DOI:** 10.1097/HS9.0000000000000868

**Published:** 2023-03-24

**Authors:** Alyx Faurel, Maël Heiblig, Olivier Kosmider, Jérôme Cornillon, Laurence Boudou, Denis Guyotat, Jean-Alain Martignoles, Yvan Jamilloux, Pauline Noyel, Elisabeth Daguenet, Anne-Camille Faure, Pierre Sujobert, Pascale Flandrin-Gresta

**Affiliations:** 1 CHU de Saint-Etienne, Laboratoire d’Hématologie, Saint-Etienne, France; 2 Hospices Civils de Lyon, Hôpital Lyon Sud, Service d’Hématologie Clinique, Pierre-Bénite, France; 3 AP-HP, Hôpital Cochin, Laboratoire d’Hématologie, Paris, France; 4 CHU de Saint-Etienne, Service d’Hématologie Clinique, Saint-Etienne, France; 5 Centre Hospitalier du Pays de Giers, Service de Rhumatologie, Saint-Chamond, France; 6 Hospices Civils de Lyon, Hôpital de la Croix-Rousse, Service de Médecine Interne, Lyon, France; 7 Hospices Civils de Lyon, Hôpital Lyon Sud, Service d’hématologie biologique, Pierre-Bénite, France

VEXAS (vacuoles, E1 enzyme, X-linked, autoinflammatory, somatic) syndrome has been recently described as a severe autoinflammatory disease associated with hematological manifestations such as vacuolization of precursors and macrocytosis.^1,2^ VEXAS is the consequence of somatically acquired mutations of ubiquitin-like modifier activating enzyme 1 (UBA1), which impair the translation of the cytoplasmic isoform of the enzyme (UBA1b). Most of the mutations described so far concern the translation initiation site of UBA1b (Met 41) directly or by altering splicing.^3^ Alternatively, 2 cases have been described with Ser56 mutation, which impairs the catalytic activity of UBA1.^4,5^

For the past 2 years, we have upgraded our diagnostic panel for hematological malignancies to include the full *UBA1* coding region (Suppl. Table S1). Here, we report 5 cases of male patients presenting with variants of uncertain significance (VUS) in *UBA1*: 1 patient (UPN-4) with a p.A478S (variant allele frequency [VAF]: 65%), 1 (UPN-2) with 2 mutations affecting the same amino acid p.D585A and p.D585E (VAF: 89% and 3%), and 3 patients (UPN-1, UPN-3, and UPN-5) with p.S621C (VAF: 80%, 85%, and 79%, respectively). Longitudinal analyses of samples were available for 2 patients (UPN-1 and UPN-2) after 5.5 and 1.5 years, respectively. The VAF of *UBA1* mutations of these 2 patients remained stable, and in the case of UPN-2, the relative contribution of the 2 UBA1 mutated clones did not change (Suppl. Figure S1). For the same 2 patients, we had access to constitutional DNA from CD3-sorted lymphocytes or skin-derived fibroblast, respectively, where the *UBA1* mutation was undetectable (Figure 1A), thus formally attesting the somatic nature of these mutations. Incidental findings of VUS raise the question of their pathogenicity, which was here supported by the following observations. First, these mutations occurred in the active adenylation domain (AAD) of UBA1, which is essential for ubiquitin activation and targeted by mutations in inherited X-linked spinal muscular atrophy (Figure 1B).^8^ Second, we observed a phenomenon of evolutive convergence with two clones with different D585 mutations in the same patient, which attest Darwinian selection because two similar independent events were selected for. Third, all these mutations modified highly conserved residues (Figures 1C),^7,8^ which further strengthen the hypothesis that these mutations are pathogenic.

**Figure 1.**
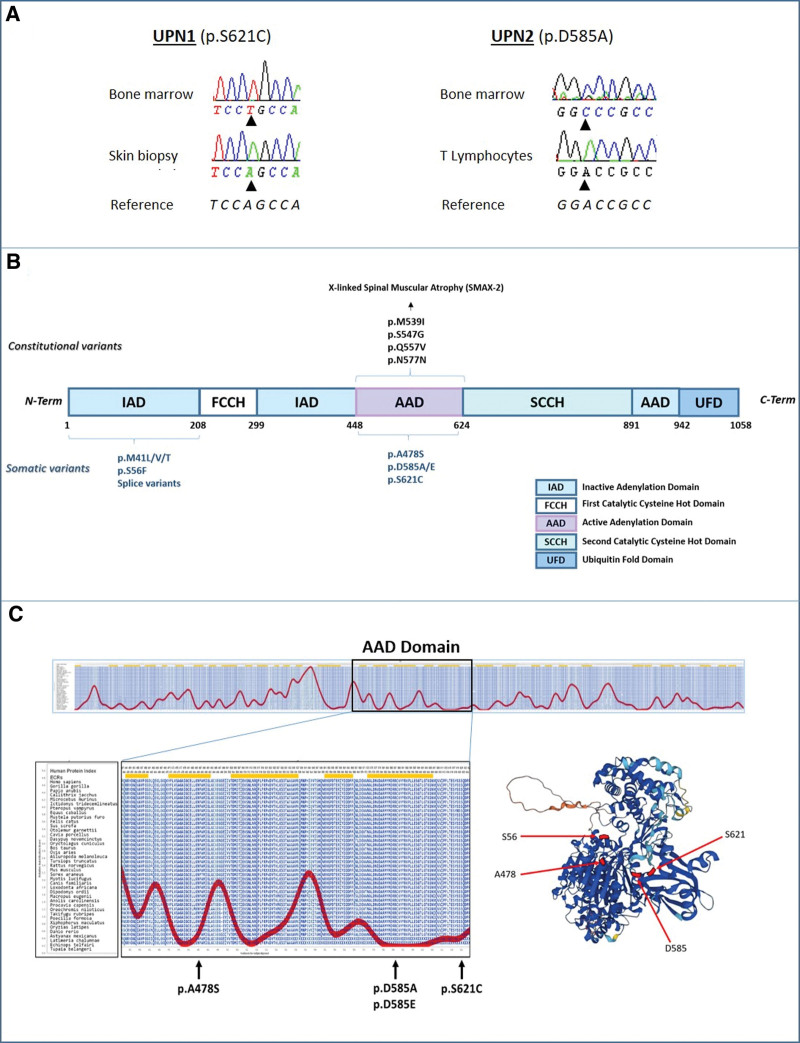
**Recurrent somatic mutations of UBA1 active adenylation domain.** (A) Sanger sequencing of UBA1 on DNA extracted from bone marrow (top) or skin biopsy or T lymphocytes from 2 different patients with mutation of the AAD domain. (B) Schematic representation of the functional domains of UBA1, and localization of constitutional (top) and somatic (bottom) mutations of UBA1. (C) Graphical representation of evolutionarily constrained regions of UBA1 and UBA1-AAD variants localization on 3D representation (Alphafold Protein Structure Database).^6,7^ AAD = active adenylation domain; UBA1 = ubiquitin-like modifier activating enzyme 1.

We then assessed the phenotype of these UBA1 AAD mutant patients by comparing them with patients reported in 3 large cohorts of VEXAS patients with UBA1 mutations affecting methionine 41.^1,9,10^

All UBA1 AAD mutant patients were males, with a median age of 69 years at the time of diagnosis. Also similarly to classical VEXAS, these patients had frequently skin lesions (nodules, neutrophilic dermatosis, and vasculitis), arthritis, and arthralgias. However, UBA1 AAD mutant patients had no or few general symptoms such as chronic fever or weight loss, lower levels of inflammatory markers, and no or few classical manifestations of VEXAS such as chondritis, pulmonary involvement, ocular involvement, or nonprovoked thrombosis (Table 1). Despite lower intensity of autoinflammatory manifestations when compared with VEXAS, 4 of 5 patients had received systemic corticosteroids for arthralgia and dermatological manifestations, which were ineffective in 1 patient, and associated with corticodependency in 2 other. Conventional disease modifying antirheumatic drugs like dapsone, hydroxychloroquine, methotrexate, mycophenolate mofetil were ineffective in 1 patient.

**Table 1:**
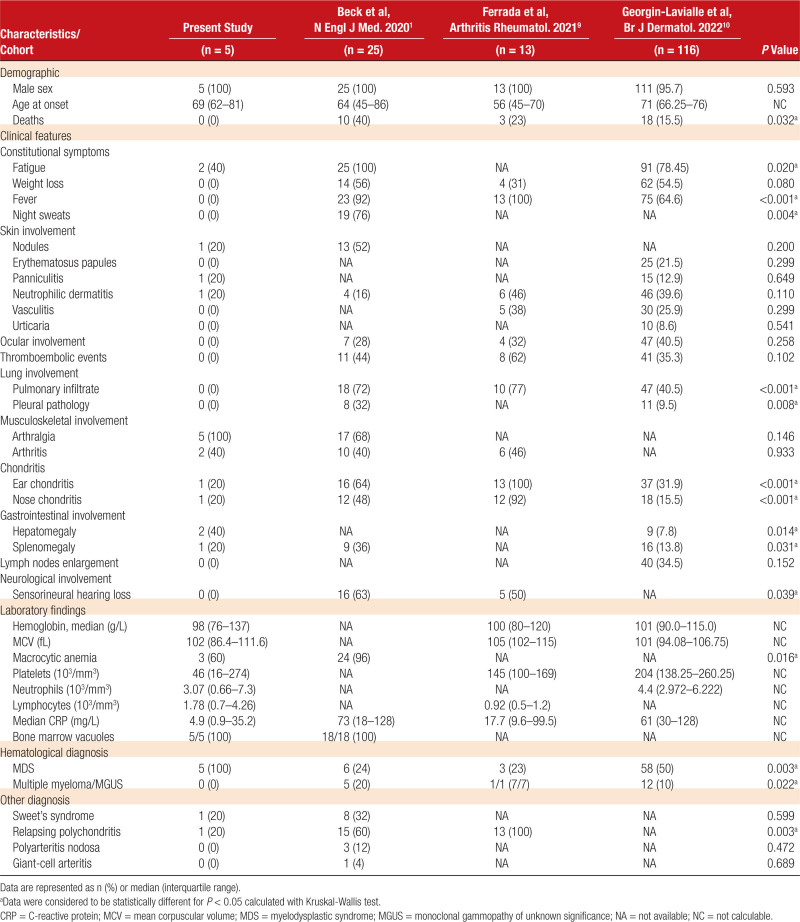
Comparison of the Clinical and Biological Data of Our Patients With 3 Large Cohorts of VEXAS Cases

UBA1 AAD mutant patients had blood count abnormalities such as macrocytic anemia (3/5, 60%), thrombocytopenia (5/5, 100%), lymphopenia (1/5, 20%), and monocytopenia (2/5, 40%) (Suppl. Figure S2A–F). Except for UPN-1 patient whose blood count parameters evolved after splenectomy, there was no significant evolution of blood parameters during follow up (mean follow up, 7.4 years; range, 2–11 years) (Suppl. Figure S3). Of note, 4 patients (UPN-1, UPN-2, UPN-3, and UPN-5) showed biochemical markers of hemolysis (increased lactate deshydrogenase activity, increased bilirubin level, and low haptoglobin level), without detectable antiglobulin antibody or schizocytes (Suppl. Figure S2G–I). Vitamine B12 levels were normal. In 3 patients with p.S621C mutant, an increased reticulocyte count was noted (918, 429, and 190 × 10^9^/L at disease onset).

Bone marrow examination lead to a formal diagnosis of myelodysplastic syndrome (MDS) in all patients, falling into the MDS with low blasts (MDS-LB, n = 4) and MDS with increased blasts type 1 (MDS-IB1, n = 1).^11^ Of note, the bone marrow was hyper cellular in all of them, as also described in VEXAS patients.^2^ However, on the contrary to classical VEXAS, UBA1 AAD mutant patients had marked erythroid hyperplasia (from 50% to 80% of total bone marrow cells). Multilineage dysplasia was noted in all patients, concerning both erythroid and megakaryocytic differentiation (n = 5; 100%) and granulocytic lineage (n = 1, 20%) (Suppl. Table S2). Ring sideroblasts were observed in low proportion (<10%) in 3 patients. Vacuolization of granulocytic and erythroblastic precursors was observed in all the patients, in a proportion of cells beyond the proposed diagnostic threshold for VEXAS syndrome (>10% of neutrophil precursors) for 4 of them (Suppl. Table S2).^12^

The p.S621C patients have a proportion of vacuolized precursors similar to that of UBA1 WT patients, whereas the other AAD-UBA1 patients (p.A478S, pD585A/E) are similar to classical VEXAS patients (Suppl. Figure S4). Cytogenetic and high-throughput sequencing of a panel of 63 genes involved in myeloid malignancies (Suppl. Table S2) were normal in 4 patients, which suggests that UBA1 AAD mutations are driving these MDSs characterized by marked erythroid hyperplasia. In the last patient (UPN-3), additional driver mutations were found (mutation of SRSF2 and NF1, and deletion of ETV6). UPN-3 was the only patient diagnosed with an overt MDS with multilineage dysplasia, associated with transfusion dependency.

Altogether, the 5 UBA1 AAD mutant patients reported here expand the spectrum of diseases associated with clonal hematopoiesis with somatic *UBA1*. As compared with canonical VEXAS patients, UBA1 AAD mutant patients have less autoinflammatory manifestations, except for skin lesions and joint disease. The hematological manifestations were partially overlapping with what is described in VEXAS patients (macrocytic anemia), but these patients were notable for having a marked erythroid hyperplasia, associated with hemolysis features in 4 of them. Interestingly, we identified previous reports of UBA1 AAD mutations with 2 cases of p.S621C in large exome series,^13,14^ and 1 mutation leading to stop codon before the AAD domain (p.Glu441Ter).^15^ Two of them had a MDS (p.S621C and p.E441Ter), while the other p.S621C patient had a granulomatosis with polyangiitis. Although these reports did not detail the clinical features of these patients, it appears that p.S621C causes a catalytic defect in the UBA1 protein confirming that the AAD domain is a mutational hot spot. After the initial description of methionine 41 mutants, the landscape of UBA1 mutations has already been enriched with splice variants leading to similar skeeping of methionine 41, but also with 2 cases of Ser56 mutants leading to impaired catalytic activity of the UBA1b isoform.^4^ AAD variants appear to be rarer than classical VEXAS variants, as we found 10 patients with p.M41 or p.S56 variants over the same study period (out of a total of 437 NGS for suspected myelodysplasias).To the best of our knowledge, UBA1 AAD mutants are the first to be associated with a specific clinical and biological presentation.

Contrary to the canonical VEXAS mutations, which impair mostly the cytoplasmic isoform of UBA1 (UBA1b), UBA1 AAD mutants are expected to affect all UBA1 isoforms in a similar manner. We can hypothesize that the mild level of autoinflammatory manifestations is due to a limited effect of these mutations on UBA1b functions, whereas the consequences of the AAD mutant on other UBA1 isoforms might explain the erythroid hyperplasia observed specifically in these patients. Beyond these differences, this study brings a new example of the clonal expansion of UBA1 mutants hematopoietic stem cells with aging, which needs to be further analyzed with functional studies. From a more practical viewpoint, we argue that *UBA1* mutations screening should not be restricted to exon 3, but should also cover the AAD coding sequence.

## AUTHOR CONTRIBUTIONS

AF, PFG, and PS designed the study and wrote the article. OK reviewed the article. MH, JC, DG, LB, JAM, and YJ reviewed the article and included patients. PN, ACF, PFG, AF, and PS were in charge of biological studies and reviewed the article. ED reviewed the article and helped with statistics.

## DATA AVAILIABILITY

Raw data are available on request to the corresponding author.

## DISCLOSURES

The authors have no conflicts of interest to disclose.

## SOURCES OF FUNDING

The authors declare no sources of funding.

## Supplementary Material


