# Supplementary material for: Recurrent Mutations of the Active Adenylation Domain of UBA1 in Atypical Form of VEXAS Syndrome
Source: Hemasphere. 2023 Mar 24;7(4):e868. doi: 10.1097/HS9.0000000000000868 (PMC10043588; doi:10.1097/HS9.0000000000000868)

**SUPPLEMENTAL DATA**

**Table S1: High throughput sequencing panel for myeloid malignancies**

| Gene name | NM |  |
| --- | --- | --- |
| ATM | NM_000051.3 | All coding sequence |
| ASXL1 | NM_015338.6 | All coding sequence |
| BCOR | NM_001123385.2 | All coding sequence |
| BCORL1 | NM_021946.4 | All coding sequence |
| BRAF | NM_004333 | All coding sequence |
| CALR | NM_004343.4 | exon 9 |
| CARD11 | NM_032415 | All coding sequence |
| CBL | NM_005188.4 | All coding sequence |
| CD28 | NM_006139 | All coding sequence |
| CEBPA | NM_004364.3 | All coding sequence |
| CSF3R | NM_156039.3 | All coding sequence |
| CUX1 | NM_181552.4 | All coding sequence |
| DDX41 | NM_016222.4 | All coding sequence |
| DNMT3A | NM_175629.2 | All coding sequence |
| EPOR | NM_000121.4 | All coding sequence |
| ETNK1 | NM_018638.5 | All coding sequence |
| ETV6 | NM_001987.5 | All coding sequence |
| EZH2 | NM_004456.5 | All coding sequence |
| FLT3 | NM_004119.3 | All coding sequence |
| GATA1 | NM_002049.4 | All coding sequence |
| GATA2 | NM_032638.5 | All coding sequence |
| GNAS | NM_001077490.2 | All coding sequence |
| GNB1 | NM_002074.5 | All coding sequence |
| HRAS | NM_004985.5 | All coding sequence |
| JAK2 | NM_004972.4 | All coding sequence |
| JAK3 | NM_000215 | All coding sequence |
| IDH1 | NM_002168.4 | exons 3 and 4 |
| IDH2 | NM_005896.3 | exon 4 |
| KRAS | NM_001130442.2 | exons 2,3,4 |
| KDM6A | NM_021140.3 | All coding sequence |
| KIT | NM_000222.3 | All coding sequence |
| KMT2A | NM_001197104.2 | All coding sequence |
| MPL | NM_005373.3 | All coding sequence |
| MYD88 | NM_002468 | All coding sequence |
| NF1 | NM_001042492.3 | All coding sequence |
| NFE2 | NM_001136023.3 | All coding sequence |
| NPM1 | NM_002520.6 | exon 11 |
| NRAS | NM_002524.5 | All coding sequence |
| PHF6 | NM_032458.3 | All coding sequence |
| PLCG1 | NM_002660 | All coding sequence |
| PPM1D | NM_003620.4 | All coding sequence |
| PTPN11 | NM_002834.5 | All coding sequence |
| PRPF8 | NM_006445.4 | All coding sequence |
| RAD21 | NM_006265.3 | All coding sequence |
| RHOA | NM_001664 | All coding sequence |
| RUNX1 | NM_001754.4 | All coding sequence |
| SF3B1 | NM_012433.4 | All coding sequence |
| SETBP1 | NM_015559.3 | All coding sequence |
| SH2B3 | NM_005475.3 | All coding sequence |
| SMC1A | NM_006306.4 | All coding sequence |
| SMC3 | NM_005445.4 | All coding sequence |
| SRSF2 | NM_003016.4 | All coding sequence |
| STAG1 | NM_005862.3 | All coding sequence |
| STAG2 | NM_001042749.2 | All coding sequence |
| STAT3 | NM_139276 | All coding sequence |
| STATB5 | NM_012448.4 | exon 16 |
| TET2 | NM_001127208.2 | All coding sequence |
| THPO | NM_000460.4 | All coding sequence |
| TP53 | NM_001126112.2 | All coding sequence |
| UBA1 | NM_003334.3 | All coding sequence |
| U2AF1 | NM_006758.2 | All coding sequence |
| U2AF2 | NM_007279.2 | All coding sequence |
| WT1 | NM_001198551.1 | All coding sequence |
| ZRSR2 | NM_005089.3 | All coding sequence |

**Figure S1 : Follow-up of UBA1 mutation variant allele frequencies**

Longitudinal analysis of samples were available for UPN-1 (p.S621C) and UPN-2 (p.D585A/E) after 5,5 and 1,5 years respectively.


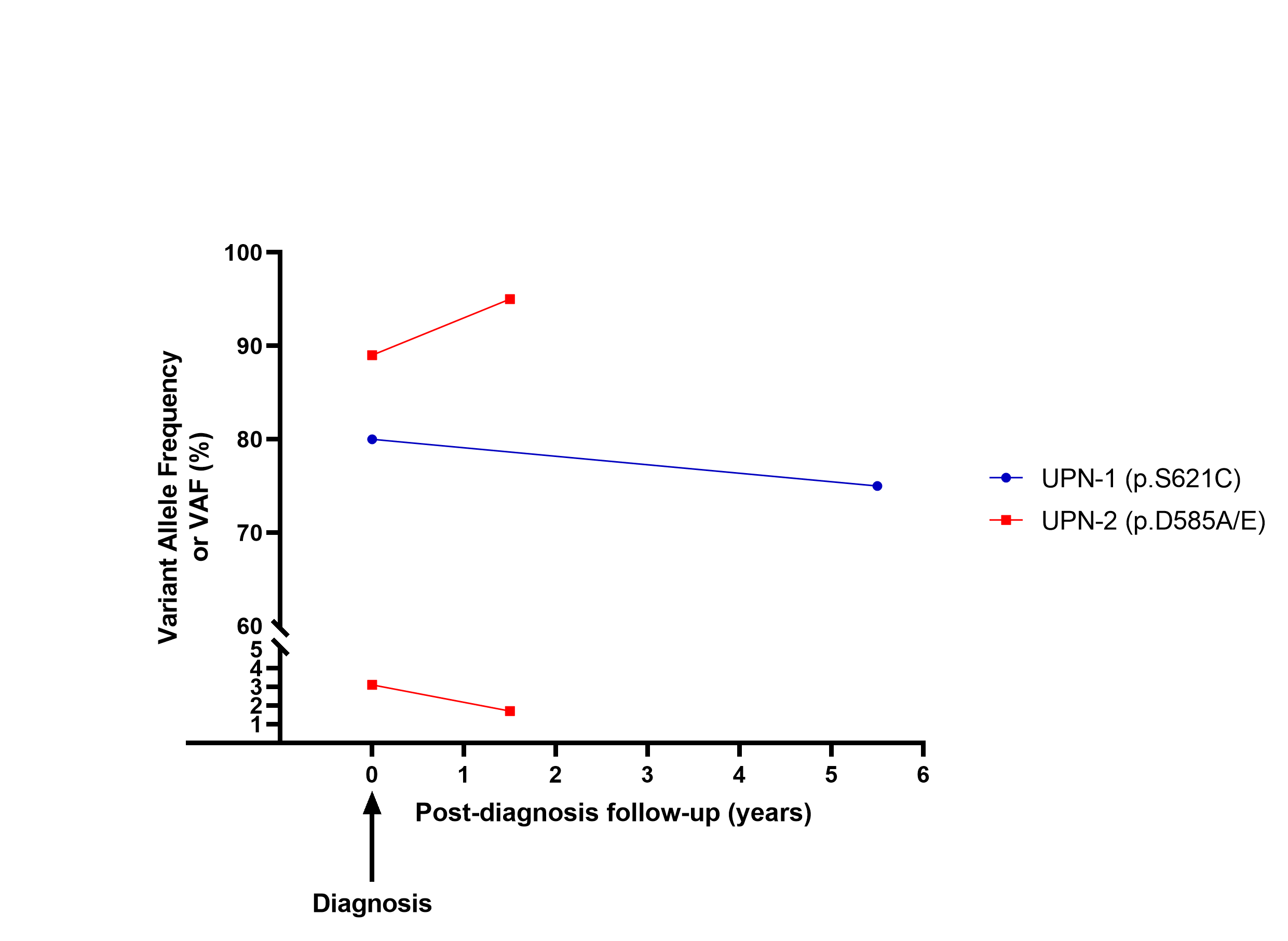


**Figure S2 : Hemogram blood test Results and biochemical markers of hemolysis**

**A-F** : Laboratory findings from blood tests performed in the last two years (2020-2022). Data are represented as n (%). MCV: Mean Corpuscular Volume, MDS : Myelodysplastic syndrome. World Health Organization (WHO) 2022 criteria were used to define cytopenia: Hemoglobin < 130 g/L, absolute neutrophil count < 1,8 G/L for leukopenia, platelets < 150 G/L for thrombocytopenia. The dotted lines represent the normal values of the studied parameters.

**G-I** : IU : International Unit, Total bilirubin (N<21µmol/L), Haptoglobin (N:0,30-2 g/L), LDH : Lactate dehydrogenase (N:135-250 IU/L). The dotted lines represent the normal values of the studied parameters.


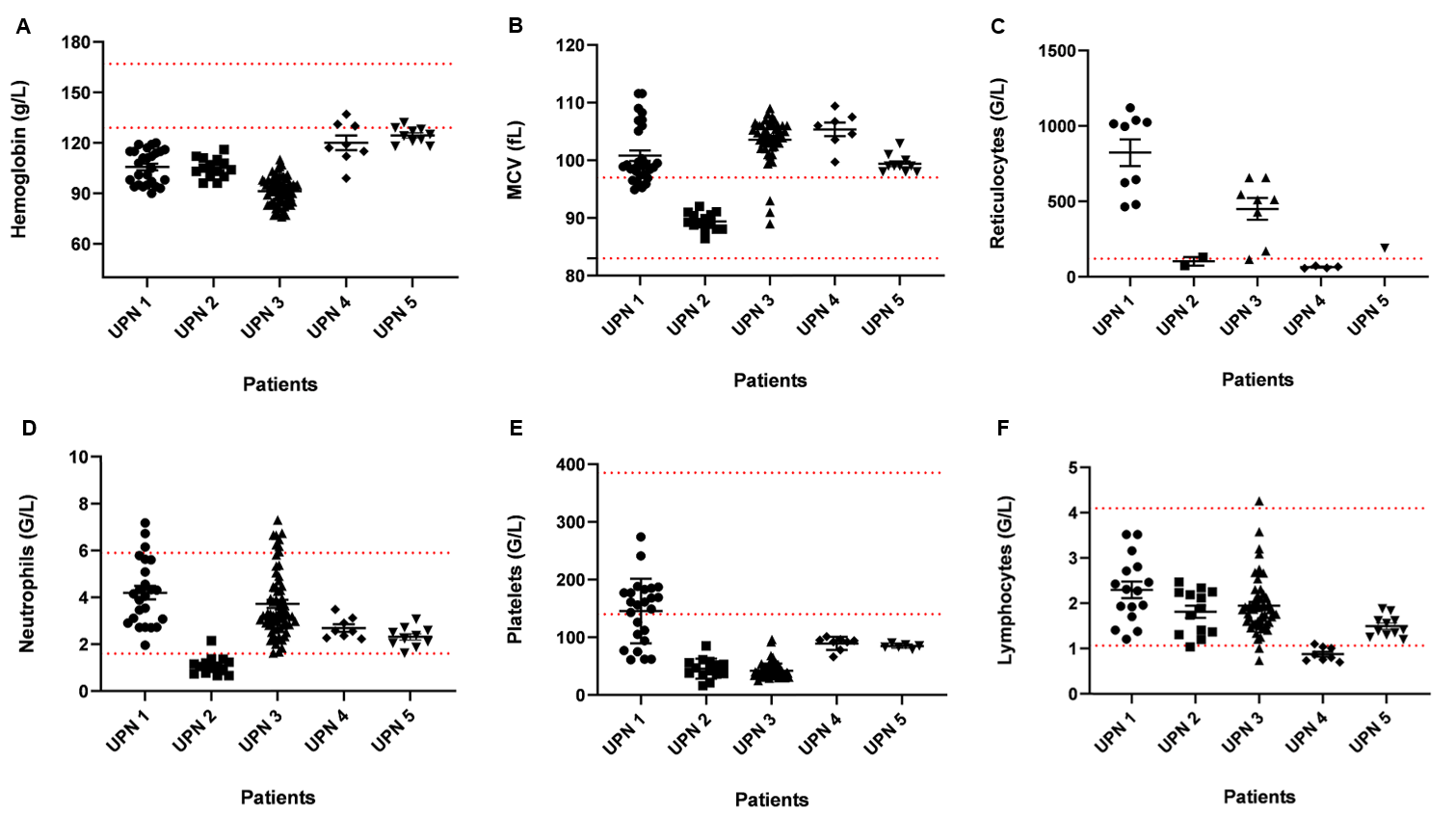


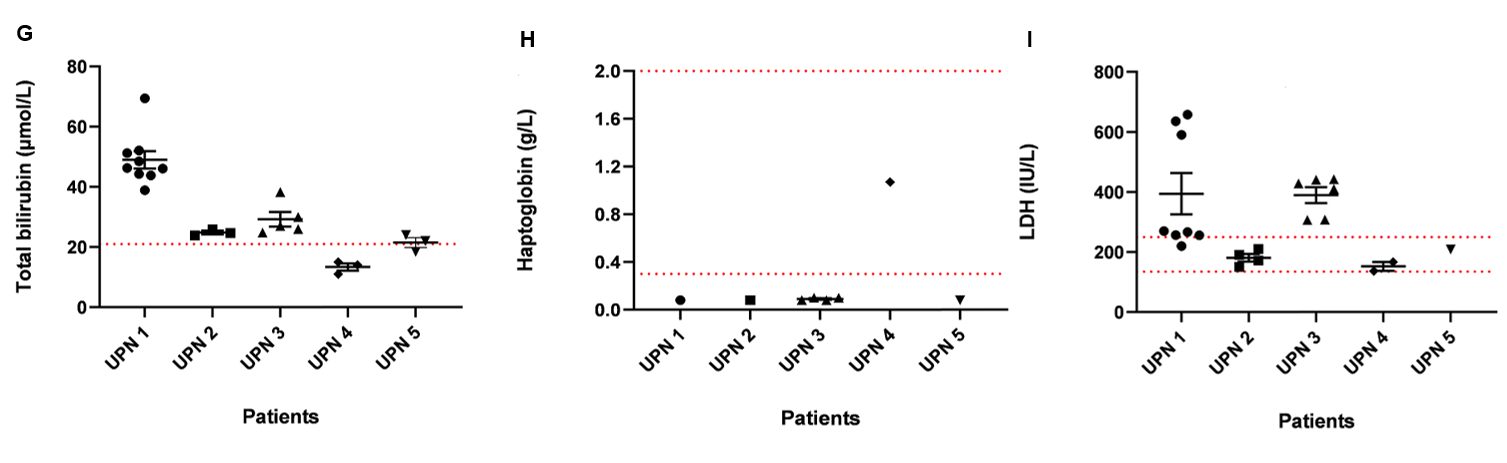


**Figure S3 : Monitoring of hematological parameters over time**

Laboratory results of blood tests performed from the first follow-up of patients until their last visit in 2022 regarding hemoglobin, mean corpuscular volume (MCV), platelets, neutrophils and lymphocytes


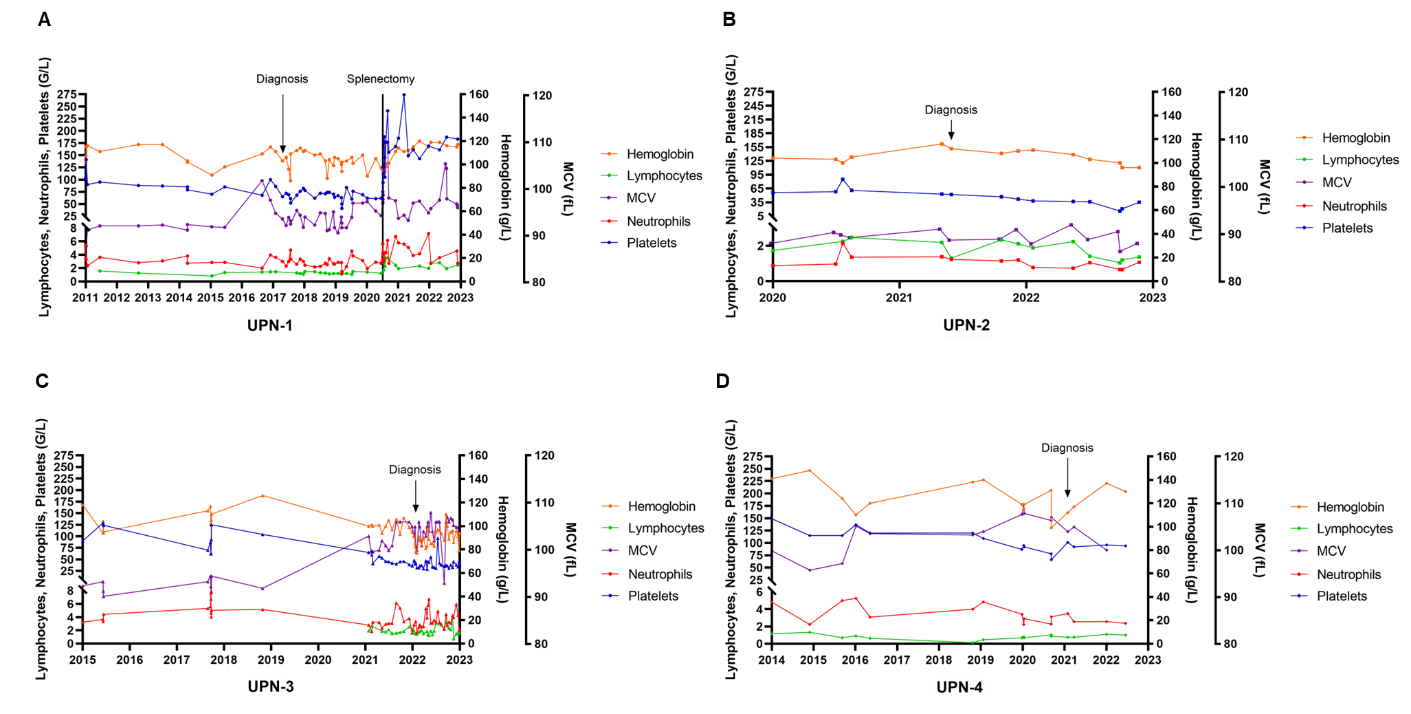


**
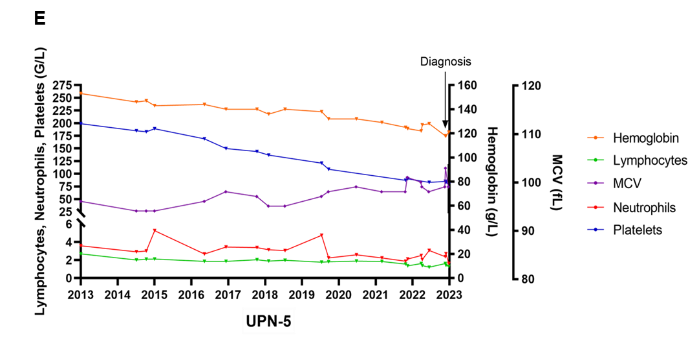
**

**Table S2: Clinical and laboratory findings of AAD-UBA1 mutant patients**

| **Characteristics / Patients** | **UPN 1** | **UPN 2** | **UPN 3** | **UPN 4** | **UPN 5** |
| --- | --- | --- | --- | --- | --- |
| **Genetics** |  | | | | |
| UBA1  mutation(s) | p.S621C | p.D585A  p.D585E | p.S621C | p.A478S | p.S621C |
| VAF at diagnosis (%) | 80 | 89  3.1 | 85 | 65 | 79 |
| Associated mutations (VAF) | No | No | SRSF2 p.P95L (32%)  NF1 p.M968K (5.2%) | No | No |
| **Clinical findings** |  | | | | |
| Constitutional symptoms | Fatigue | No | Fatigue | No | No |
| Skin involvement | Sweet syndrome | No | No | Lupus timidus | No |
| Musculoskeletal involvement | Arthritis  Arthralgia | Arthritis  Arthralgia | Arthritis  Arthralgia | Arthralgia | Arthralgia |
| Chondritis | Yes | No | No | No | No |
| Hepatomegaly | Yes | No | No | No | Yes |
| Splenomegaly | Yes  (Splenectomy in 2020) | No | No | No | No |
| Others | Hypertension  angina with exertional dyspnea,  Sleep apnea syndrome. | Hypertension,  Edema of the lower limbs,  Gout  Gastric ulcer. | Hypertension,  Diabetes  Reynaud's disease,  Right partial nephrectomy,  Sleep apnea syndrome. | Primary syphilis | AV block,  Benign prostatic hyperplasia,  Gastritis,  Colonic adenoma  Sleep apnea syndrome. |
| **Hematological blood findings** |  | | | | |
| Macrocytic anemia | Yes | No | Yes | No | Yes |
| Thrombocytopenia | Yes | Yes | Yes | Yes | Yes |
| Reticulocytes count at onset (G/L) | 918 | 74 | 429 | 67 | 190 |
| **Bone marrow** |  | | | | |
| Hypercellularity | Yes | Yes | Yes | Yes | Yes |
| Ratio M:E | 0,2:1 | 0,2:1 | 0,5:1 | 0,5:1 | 0,5:1 |
| Megakaryocyte dysplasia | >10% | >10% | >10% | >10% | >10% |
| Erythroid dysplasia | >10% | >10% | >10% | >10% | >10% |
| Myeloid dysplasia | No | No | >10% | No | No |
| Vacuolized erythroid precursors (%) | 7,70 | 35,48 | 4 | 24 | 6,3 |
| Vacuolized myeloid precursors (%) | 30,95 | 26,32 | 3 | 37 | 11 |
| **Karyotype** | 46,XY | 46,XY | 46,XY,del(12)(p12p13) [16/20] ; 46,XY[4/20] | 46,XY | 46,XY |

**Figure S4 : Graphical distribution of AAD-UBA1 patients (yellow dots) compared to classical UBA1 mutated patients (red dots) and UBA1-WT patients (blue dots) according to their proportions of vacuoles in erythroid (Y-axis) and myeloid precursors (X-axis).**


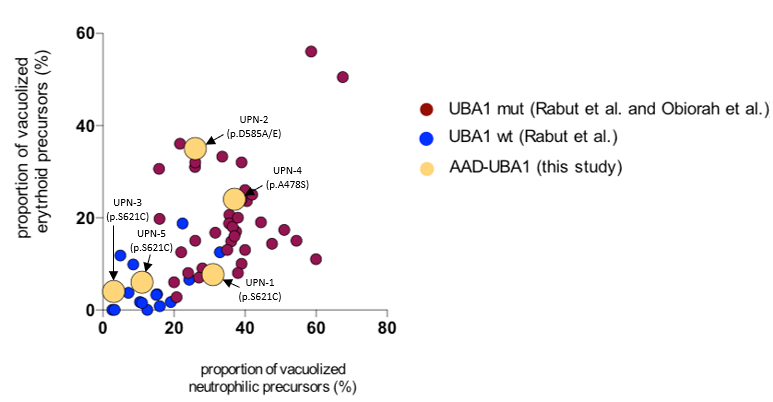

Supplement: Supplementary file 1 [file hs9-7-e868-s001.docx]
